# Splenic Torsion in a 16-Year-Old Female With a Wandering Spleen: A Case Report

**DOI:** 10.7759/cureus.111195

**Published:** 2026-06-20

**Authors:** Ghaith M Bani Abdel-Rahman, Laith H Halasah

**Affiliations:** 1 General Surgery, Ministry of Health, Amman, JOR

**Keywords:** congenital anomalies of the spleen, emergent splenectomy, splenic torsion, surgical acute abdomen, wandering spleen

## Abstract

Wandering spleen is a rare clinical condition characterized by excessive mobility of the spleen due to laxity or absence of its supporting ligaments. It may present with non-specific abdominal symptoms or an acute abdomen and peritonitis secondary to torsion of the splenic vascular pedicle. In this article, we report the case of a 16-year-old female who presented to the emergency department with acute abdominal pain and was diagnosed with splenic torsion on contrast-enhanced computed tomography. Prompt surgical intervention was undertaken, resulting in a favorable outcome. This case highlights the importance of early recognition and management of this uncommon but potentially life-threatening condition.

## Introduction

Wandering spleen is an uncommon entity, accounting for fewer than 0.2% of splenectomies [1]. It results from congenital or acquired laxity of the splenic suspensory ligaments, including the gastrosplenic, splenorenal, and splenocolic ligaments [1-4]. This condition predisposes the spleen to abnormal mobility within the abdomen, increasing the risk of torsion of the splenic pedicle, which may lead to infarction [3]. Although it can occur at any age, it is more frequently observed in pediatric populations and women of reproductive age [5,6]. Clinical presentation varies widely, ranging from incidental findings to acute abdomen, and a mobile abdominal mass can be palpated in some cases [1-4]. Imaging, particularly contrast-enhanced computed tomography (CT), plays a critical role in diagnosis. Management depends on splenic viability and includes splenopexy or splenectomy [7].

## Case presentation

A 16-year-old previously healthy female presented to the emergency department with a five-day history of progressively worsening abdominal pain. The pain was initially localized to the left paraumbilical region, but later the pain became more generalized. It was described as constant, dull, and associated with nausea without any change to the patient’s bowel habits. She denied fever, urinary symptoms, or recent trauma. On examination, the patient appeared uncomfortable but was hemodynamically stable with a pulse rate of 110 beats/minute, blood pressure of 101/63 mmHg, temperature of 38°C, and pulse oximetry of 94% on room air. Abdominal examination revealed generalized tenderness over the entire abdomen and guarding with rebound tenderness over the left paraumbilical region. Bowel sounds were present.

Laboratory investigations showed leukocytosis (white blood cell count: 18,200/µL) with a neutrophilic predominance and a low hemoglobin of 9.9 g/dL. Platelet count and serum chemistry were within normal limits. A bedside ultrasound scan showed large splenomegaly reaching the left iliac fossa and the pelvis, with mild intra-abdominal free fluid.

Given the atypical presentation, a contrast-enhanced CT scan of the abdomen and pelvis was performed. Imaging revealed absence of the spleen in its normal anatomical location in the left upper quadrant (Figure 1). Instead, the spleen was identified as lying low in the left mid-abdomen. The spleen appeared enlarged, measuring about 24 cm in its craniocaudal dimension, reaching the pelvis, with poor enhancement and a “whirl sign” of the splenic vessels, consistent with torsion of its vascular pedicle (Figure 2). Findings were highly suggestive of splenic torsion in the setting of a wandering spleen.

**Figure 1:**
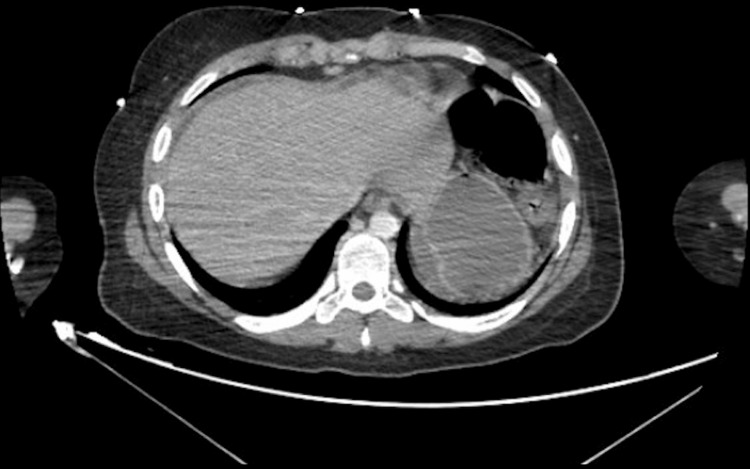
Computed tomography scan showing the left upper quadrant of the patient’s abdomen without the spleen.

**Figure 2:**
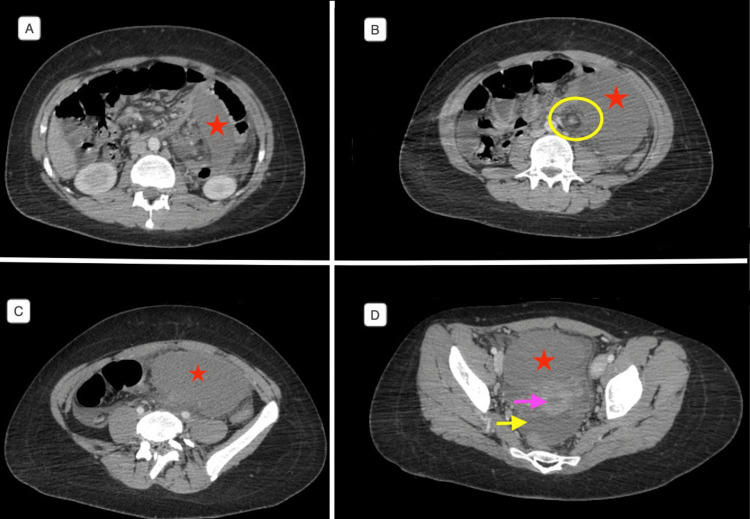
Computed tomography scan with the wandering spleen marked with a red start throughout all images. (A) Wandering spleen positioned in the left lumbar region. (B) “Whirl sign” of the splenic vascular pedicle inside the yellow circle. (C) The wandering spleen reaching the spleen with poor attenuation. (D) The wandering spleen reaching deeper into the true pelvis, marked by the red star; the uterus is marked by the tip of the pink arrow, and free pelvic fluid is noted at the tip of the yellow arrow.

After initial fluid resuscitation in the emergency room, the patient was taken to the operating room for exploratory laparotomy, which was performed through the midline starting about 4 cm above the umbilicus and extending about 4 cm inferior to it. Intraoperatively, the spleen was found in the left lumbar region with a 720-degree clockwise torsion of the splenic pedicle. The spleen appeared congested and non-viable (Figure 3). Given these findings, a splenectomy was performed, and washing and suctioning of the abdominal cavity were done, followed by closure of the laparotomy wound.

**Figure 3:**
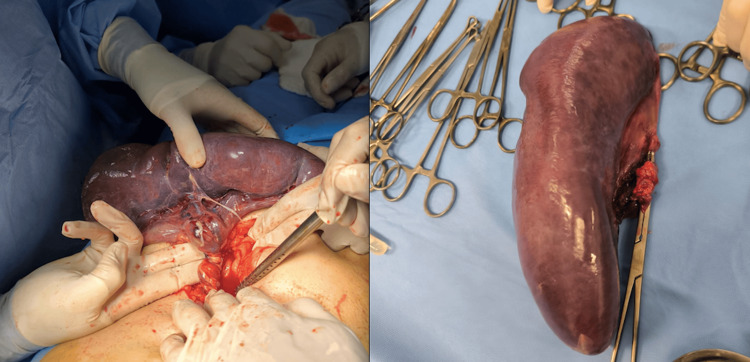
Intraoperative images of the wandering spleen showing its twisted vascular pedicle on the left, and the spleen after being removed on the right.

The postoperative course was uneventful. She was discharged on postoperative day four in stable condition with instructions for follow-up. The patient received appropriate vaccinations against encapsulated organisms two weeks after her surgery. Histopathological examination confirmed splenic infarction without evidence of malignancy.

## Discussion

Wandering spleen is a rare but important differential diagnosis in patients presenting with abdominal pain and a palpable abdominal mass [1]. The condition arises due to congenital absence or laxity of splenic ligaments, though acquired factors such as trauma, hormonal changes, or connective tissue disorders may also contribute [3]. Splenic torsion is the most serious complication and may result in vascular compromise, infarction, and potential rupture [1-4]. Clinical presentation is often non-specific, which can delay diagnosis. Intermittent torsion-detorsion events may cause chronic or recurrent abdominal pain, while complete torsion leads to acute abdomen [3]. Contrast-enhanced CT is the imaging modality of choice. Hallmark features include absence of the spleen in its normal position, ectopic splenic location, splenomegaly, poor or absent enhancement, and the “whirl sign” indicating twisted splenic vessels [7]. Management depends on splenic viability. In viable spleens, splenopexy is preferred, particularly in pediatric patients, to preserve immunologic function [5,6]. However, in cases of infarction or non-viability, splenectomy is indicated, as in our patient [2,3]. Post-splenectomy patients are at risk for overwhelming post-splenectomy infection, necessitating appropriate vaccination and patient education [4].

## Conclusions

Splenic torsion due to a wandering spleen is a rare but potentially life-threatening condition that should be considered in young patients presenting with atypical abdominal pain and a mobile abdominal mass. Early diagnosis using contrast-enhanced CT and prompt surgical intervention are crucial to prevent complications. Preservation of the spleen should be attempted when feasible; however, splenectomy remains necessary in cases of infarction. Awareness of this condition can facilitate timely management and improve patient outcomes. The current literature is scarce regarding this rare entity, and more case series are needed to better understand the signs and symptoms of this anomaly and enhance the clinical approach to its management.
